# ‘Once upon a time …’: Orphanhood, childhood studies and the depoliticisation of childhood poverty in southern Africa

**DOI:** 10.1177/0907568215589419

**Published:** 2015-06-12

**Authors:** Nicola Ansell

**Affiliations:** Brunel University, UK

**Keywords:** Agency, AIDS, orphanhood, poverty, southern Africa

## Abstract

Policy, interventions and research concerning southern African children remain dominated by a focus on AIDS-related orphanhood, although the association between orphanhood and disadvantage is highly questionable. I argue that the trope of the AIDS orphan serves a range of agendas, including for academic research. In particular, orphans represent the quintessential child-agent, celebrated in fairytales and fiction. Finally, I examine how this has led to a policy response – education bursaries – that cannot adequately address childhood poverty in the region.

Over the past decade, public policy and non-governmental organisation (NGO) interventions relating to children in southern Africa have coalesced around a population of ‘orphans and vulnerable children’, self-evidently produced by the region’s AIDS pandemic. This policy focus is paralleled in burgeoning academic research on the impacts of AIDS and various dimensions and outcomes of orphanhood (work on child-headed households, young carers and so on). Such a perspective is recognised to be problematic because it neglects the structural poverty that affects both orphans and many other children (Bray, 2003; Meintjes et al., 2010). Yet, I argue in this article, childhood studies research, in emphasising children’s agency, is complicit in perpetuating the focus on orphanhood.

I begin by presenting evidence that contests the significance of orphanhood in determining disadvantage, and then move on to explore why AIDS orphans have been afforded such attention in both policy and research. I explore how the focus on orphans prefigures the adoption of educational bursaries as a solution – a solution that fails both to mitigate any individual disadvantage experienced by AIDS orphans and to address the structural poverty that shapes the lives of a much greater number of southern African youth. I conclude by advocating that childhood studies might usefully adopt a social justice lens, although this could require an alternative methodological approach.

## Orphanhood and individual disadvantage: A dearth of evidence?

### Livelihoods research in Malawi and Lesotho

In 2007 and 2008, I undertook, with colleagues,^1^ a research project in Malawi and Lesotho that sought to explore the processes through which AIDS impacts on food insecurity. De Waal and Whiteside (2003) had proposed that recurrent food crises in a number of southern African countries were associated with high HIV prevalence. The proposed mechanisms strongly related to the impacts of the disease on young people. AIDS orphans, it was suggested, may fail to inherit land or other productive assets, and transmission of knowledge and skills between the generations may be disrupted, leaving young people ill-prepared to build food-secure livelihoods for themselves.

Our research set out to examine these mechanisms among young people in one rural village in each country. We surveyed all households, collecting individual and household level data. Based on this data, in each village we selected a broadly representative sample of approximately 40 young participants, more than half of all those aged 10–24. Half of the participants had experienced the long-term chronic illness and/or premature death of a close adult family member^2^ (we categorised these as ‘AIDS-affected’) and half had not (these were categorised as ‘unaffected’). The young people participated in nine research activities, most of which involved them in self/group-directed production of a diagram, visual or dramatic output. The majority of older youth (aged 18–24) from both villages, irrespective of whether they had participated in the initial research activities, subsequently took part in life history interviews.

The research very quickly confounded our expectations that there would be observable differences between the young people we defined as ‘AIDS-affected’ and those we defined as ‘unaffected’. We had anticipated that those affected by AIDS would more likely have left school prematurely to care for relatives, work for their households or to earn an income. Equally, we expected AIDS-affected young people to lack the skills or resources required for more lucrative occupations. None of these expectations held true. While our research was certainly not statistically representative, we were struck by the absence of any clear relationship between being ‘AIDS-affected’ and individual disadvantage in accessing livelihood opportunities.

Agricultural land in both communities was scarce. While a minority of young people had their own fields, few had sufficient land to support a livelihood. Although orphanhood had an impact, it was one among many factors shaping access to land and did not operate in a predictable way to deprive young people of their inheritance. In neither country is land generally transferred only at the point at which the land-holder dies. Practices differ between the two contexts and gender is a key determinant. In Lesotho, fields are traditionally allocated to a man when he marries, but today household land is usually passed entirely to the eldest son. Despite recent legal changes, very few women held land in their own right. In Malawi, by contrast, girls are often allocated a small field before marriage, and men generally need to marry to access land through their wives. The effects of AIDS were not clear-cut. While some young people complained they had been denied access to their parents’ land when they were orphaned, ostensibly because they were too young to make use of it, others had acquired land prematurely, and found themselves with access to a resource that most of their contemporaries lacked.

Similar stories were told about accessing other resources. Where parents had engaged in artisanal activities, some children told of their struggles to maintain ownership of equipment – struggles that sometimes failed. In general, however, very few young people had access to productive resources, irrespective of their family status.

In terms of accessing livelihood skills and knowledge, most young people said that they learned skills not from parents but from peers. Moreover, most children who could not reside with parents stayed with grandparents, aunts, uncles or stepparents and were certainly not spared the opportunity to engage in agricultural and other forms of work to support their households. They doubtless picked up skills they might otherwise have acquired by working with and for their parents.

Not only was it impossible to identify generalised disadvantages among the AIDS-affected young people in terms of their access to livelihood resources; livelihood outcomes too were indistinguishable from those of unaffected youth. In Malawi, roughly equal numbers of affected and unaffected young people were engaged in small businesses, in irrigated (and therefore more lucrative) agriculture and in casual work. In Lesotho, affected and unaffected boys were equally likely to be employed herding.

The only striking distinction between the two groups, particularly in Lesotho, was that AIDS-affected young people were more likely to attend school (Table 1). Although some reported having left school upon the death of a parent (usually a father), this was but one among many factors that precipitated school dropout. Among 18- to 24-year-olds, those deemed AIDS-affected had on average progressed further through school in both villages (young men in Lesotho by a full 4 years). Of the 14 young people from Ha Rantelali studying elsewhere, 10 were AIDS-affected (Ansell et al., 2014). These findings cannot be generalised, but do appear to corroborate other studies suggesting orphanhood affects school attendance less negatively than was once believed (Ainsworth and Filmer, 2006). I return to examine the reasons for this discrepancy in a later section.

**Table 1.** Numbers/percentages of young people regularly attending school in the case study villages.

Ha Rantelali, Lesotho			
10- to 17-year-olds	Affected	22/27	81%
10- to 7-year-olds	Unaffected	17/25	68%
18- to 24-year-olds	Affected	5/16	31%
18- to 24-year-olds	Unaffected	0/13	0%
Nihelo, Malawi			
10- to 17-year-olds	Affected	10/12	83%
10- to 17-year-olds	Unaffected	19/22	86%
18- to 24-year-olds	Affected	1/16	6%
18- to 24-year-olds	Unaffected	1/24	4%

### Other research findings

While novel in examining impacts on livelihoods, ours is not the only empirical research to question the association between AIDS orphanhood and individual disadvantage. Sherr et al. (2008) undertook a systematic review of literature on the impacts of orphanhood. While they found most studies reported some negative effects across a wide range of physical, socioeconomic and psychological outcomes, there were often no differences detected. Parikh et al. (2007) in their South African cohort study found no statistically significant differences in most education, health and labour outcomes between orphans and the non-orphans with whom they lived, although paternal orphans were more likely to be behind in school. Evidence from the Young Lives Ethiopian study reveals little difference in school enrolment, school attendance or body mass index between orphans and non-orphans, and where small differences exist, they sometimes favour orphans (Crivello and Chuta, 2012). Similarly, in South Africa, Tamasane and Head (2010) found very little difference in the quality of material care provided by grandparents and other carers, including biological parents, a situation that might be attributable to provision of old age pensions. Even at a macro-level, Young (2005) has calculated that the economic impact of AIDS in South Africa is likely to be positive, because its effect on fertility rates outweighs any loss of human capital associated with school dropout among orphans.

Relatedly, research has confounded common misconceptions about AIDS and orphanhood. Meintjes and Giese (2006) point to the fact that fewer than half of non-orphans in South Africa live with both parents. Similarly, Crivello and Chuta (2012) note that more Ethiopian children are separated from parents by other causes than by orphanhood. Moreover, 85% of Africa’s orphans have a surviving parent (Meintjes and Giese, 2006). Thus, orphans’ living arrangements are not necessarily very different from those of non-orphans. Furthermore, high levels of orphanhood are not new in Africa; Campbell et al. (2010) observe that of 45 million orphans, only 11.4 million are attributable to AIDS. In Ethiopia, as few as 20% of orphans have lost parents to AIDS (Crivello and Chuta, 2012). Similarly, Meintjes et al. (2010) show that child-headed households account for only 0.47% of South African households. Of the children living in these, 92.1% have a living parent. The fact that not all child-headed households are headed by orphans has also been noted in the Namibian context (Ruiz-Casares, 2009).

Henderson (2006) has highlighted how focusing on the vulnerabilities of AIDS orphans obscures similarities between their circumstances and those of other poor children. Examining published household surveys in South Africa, Richter and Desmond (2008) found neither orphans nor those in child-only households were the worst-off children. This observation is echoed by many other researchers. Campbell et al. (2010) note that ‘Gender and region of residence are much more important predictors of poor schooling outcomes [than orphan status], and for all outcomes household wealth is the single most important correlate of better outcomes’ (p. 12). In Ethiopia, poverty and household location account for much larger differences in education and health indicators than orphan status (Crivello and Chuta, 2012). For Abebe (2010), ‘rather than the lack of biological parents it is the combination of the absence of a carer and the presence of acute poverty and economic marginality that explain various forms of vulnerability in orphans and non-orphans’ (p. 540). And as Meintjes and Giese (2006) note, at neighbourhood level, orphanhood is not necessarily considered the main indicator of children’s vulnerability. Campbell et al. (2012) even suggest that for children in Zimbabwe, stigma related to AIDS may be less problematic than stigma related to poverty.

Some scholars have observed that by obscuring the effects of poverty, focusing on AIDS orphanhood is positively harmful. Crivello and Chuta (2012) remark,

OVC [orphans and vulnerable children] is not simply an innocuous bureaucratic label created to measure parental death and child vulnerability across diverse contexts; it also shapes thinking about who the world’s vulnerable children are, and to funnel global funds to support them. (p. 546)

Ainsworth et al. (2005) noting that Tanzanian children’s school attendance became less regular in the months preceding an adult household member’s death, recommended generalised improvements in school quality and access to secondary schooling, and using targeted strategies only to address specific constraints faced by AIDS-affected children (time constraints and psychological impacts). Currently, funding is often misallocated. Not only do poor non-orphans fail to benefit; orphans, too, may be harmed. Meintjes and Giese (2006) observe that linking orphans to material resources that are not available to others ‘commodifies’ their orphan status. If a child becomes a ‘new terrain to access scarce resources’ (Reynolds et al., 2006: 298) for a household, this can make them more vulnerable.

The discourse around orphanhood has not remained entirely static. Rather than AIDS orphans, most organisations now refer to ‘orphans and (other) vulnerable children’ (OVC). The last ‘Children on the Brink’ report (UNICEF/UNAIDS, 2004) clearly stated that targeting interventions only at orphans was inappropriate, but this failed to shift the focus of many policies and interventions towards a wider group of children (Meintjes and Giese, 2006). Moreover, while AIDS is no longer directly referred to in the term ‘orphans and vulnerable children’, ‘Orphans are conspicuously the only named category of children, and despite disentangling itself in name from the AIDS epidemic, it remains strongly rooted in the global AIDS agenda’ (Crivello and Chuta, 2012: 538).

## Explaining the focus on orphanhood: What agendas are served?

The attention given to orphans in the international child protection discourse suggests that orphanhood is a major, if not the major factor affecting child vulnerability in sub-Saharan Africa. (Crivello and Chuta, 2012: 537)

A growing body of research recognises that this is not the case, but little attention has been paid to the reasons for the singular focus on AIDS orphanhood. I suggest below a number of possible explanations, and some of the international and national agendas that are served by the trope of the AIDS orphan.

### Western common sense expectations: Orphanhood must make a difference

From a Western perspective, alarm at the scale and possible consequences of orphanhood is unsurprising. In the early 2000s, a third or more of adults in some African countries were believed to have contracted a disease that would kill them within a decade. Many children would be left with neither biological parent. Although orphanhood had been relatively common in poorer countries, the spectre of the AIDS pandemic constructed it as an issue of global concern.

Orphanhood is particularly concerning to Western society for several reasons. First, in the West, nuclear families have long predominated and are viewed as the ‘natural’ milieu for child rearing. Where almost all children live with at least one parent, parents are viewed as indispensable to the successful raising of children. Second, attachment theory – the idea that every child requires a near-exclusive relationship with a mother or ‘permanent mother-substitute’ for its first few years – has been influential since the mid-20th century. Third, both folklore and research (Akerman and Statham, 2011; Kimball, 1999) suggest that, in Western societies, orphanhood is a significant marker of disadvantage. While each of these phenomena may apply in Western society, they do not translate globally. In many societies, children are raised in extended families by a more diffuse group of adults, various relatives participating in ‘parenting’ even where both biological parents are living.

### The ‘quintessential vulnerable child’: A focus for child-saving

While in some respects concern with the figure of the orphan reflects its incongruity with Western assumptions of appropriate childhood,^3^ it also serves a functional role. The AIDS orphan has become the ‘quintessential vulnerable child’ (Meintjes and Giese, 2006: 408), following a line of others (street children, trafficked children, child soldiers), that justifies a ‘child saving’ mission. As Meintjes and Giese (2006: 408) point out, ‘International agency advocacy and intervention, government policy and practice, service design and provision, media reporting, popular discourse and responses from concerned citizens all draw on the idea of the orphan’. Orphans are particularly amenable to a discursive construction as vulnerable, as they seemingly lack parental protection and may be represented as isolated and wholly dependent on the pity of external providers. The emotive image of the orphan justifies action and mobilises funding (Meintjes and Giese, 2006). Work with orphans provides photo opportunities for celebrities and a clear and worthy purpose to children-focused NGOs. Orphans can readily be viewed as innocent victims – they cannot be implicated in their own situation, which offers an uncomplicated story (one that casts blame, if anywhere, on adults in their own families rather than on causes susceptible to policy solutions). Researchers working with children living on the streets in the 1990s warned that categorising children according to one aspect of their lives de-contextualised them from the wider social and political environment (Bray, 2003). De-contextualisation, however, serves the interests both of fundraisers who want simple stories to present, and policy makers who like one-size-fits-all approaches that can be applied continent-wide. Thus, UN agencies and NGOs employ orphanhood to maintain donors’ attention to the social and economic consequences of AIDS (Bray, 2003).

### Looming chaos: Fear of social disorder

The motivation for focusing on AIDS orphans relates not only to their appeal as a humanitarian cause but also reflects fear of the potential social consequences. UNICEF’s 2003 report on *Africa’s orphaned generations*, for example, emphasises that ‘The orphan crisis in sub-Saharan Africa has implications for stability’ (p. 43), explaining that children may react to stress through aggressive and anti-social behaviour, and arguing that the crisis may compromise countries’ development prospects. Bray (2003) has described the chain of causality through which South African society envisages the production of a generation of anti-social children that will precipitate a breakdown in the social fabric. This moral panic around AIDS orphans is fuelled by the position of children in South African society and norms around social control (Bray, 2003).

### Intersections of children and AIDS: A funding magnet

Attention to AIDS orphans is also associated with the scale and nature of funding available for interventions relating to AIDS, in comparison to funding for social issues more generally. The US government-funded President’s Emergency Plan for AIDS Relief (PEPFAR) spent over $5 billion on bilateral HIV/AIDS programmes in 2012 (PEPFAR, 2013). The Global Fund, a largely government-funded international financing institution that works predominantly on AIDS, disbursed $2.7 billion in 2011 (Global Fund 2012). The Bill and Melinda Gates Foundation (2012) spent $219 million of their Global Health budget on AIDS in 2011.^4^ Many other multi-lateral and bilateral donors, UN agencies, NGOs and philanthropic organisations also devote substantial funds to AIDS. Work with orphans holds a particular appeal to these organisations. While HIV prevention interventions can be highly controversial, and many aspects of AIDS remain sensitive or stigmatised, donors, NGOs and governments are generally happy to be associated with child-saving. Detailed data on funding allocations are difficult to obtain, but PEPFAR sets aside 10% of its programme funding to address the needs of orphans and vulnerable children and claims to have supported more than 5 million of them thus far (PEPFAR, 2014). Similarly, the Global Fund (2012) assisted 6.2 million OVC over its first decade to 2012.

Not only are children appealing to funders of AIDS projects, but those working with children see the funding attached to the theme of HIV and AIDS as a valuable resource. Much work with children in Africa is thus refracted through a lens of ‘OVC’ in order to secure funding (see Cheney, 2010b). UNICEF, for instance, made HIV and AIDS one of five focus areas in its 2006–2013 strategic plan and this remains one of nine funding pools in the 2014–2017 plan. It spent $102 million of its $3 billion core budget on HIV/AIDS and children in 2012, of which a quarter was dedicated to supporting ‘national capacity to increase the proportion of children orphaned or made vulnerable by HIV and AIDS receiving quality family, community and government support’ (UNICEF, 2013: 35). Bray (2003) has questioned whether UNICEF has promulgated emotive imagery of orphans in order to secure funding.^5^

### Neoliberal agendas

Funding of interventions for AIDS orphans relates not only to their popular appeal, but also serves the political interests of donors committed to neoliberal agendas. Both childhood and the aid industry are depoliticised in popular discourse although beneath the surface both are highly political (Cheney, 2010a). Highlighting orphans deflects attention from the structural roots of poverty. As Cheney (2010a) points out, ‘Focusing on enumerating the numbers of individual vulnerable children in this context becomes a way to avoid tackling the bigger issues of poverty and structural violence that affects entire populations’ (p. 6). The ‘problem’ of AIDS orphans is defined as a technical rather than a political issue (viz. Ferguson, 1990), and one best handled by NGOs that enthusiastically compete for pools of funding in order to implement their chosen interventions (see Ansell, 2010). Both international and local organisations produce hybrid representations of children as ‘individuals in need of saving, of developing personal autonomy, or of exercising individual rights’ (Ansell, 2010: 791) that unintentionally serve neoliberal agendas. Such competition between non-state service providers serves donors’ ideological interests. National OVC policy becomes an exercise in attracting funding and coordinating NGO and donor activities, rather than delivering services (Cheney, 2010b).

Responses to AIDS orphanhood, therefore, do not engage with systemic causes of poverty. In line with neoliberal thinking, responsibility for poverty and disadvantage is located in individuals and families rather than social, economic or political processes, and Western governments or society are certainly not implicated. Moreover, rather than transforming the structures that make people vulnerable, solutions are seen to lie in the appropriate targeting of individuals. Before undertaking our field research in Lesotho and Malawi, we consulted potential ‘stakeholders’ – representatives of government, UN agencies and NGOs. These stakeholder groups were particularly concerned that our research findings should help them with targeting.

As Meintjes and Giese (2006) have pointed out, most programmes targeting orphans actually focus on alleviating their poverty. However, only a minority of poor children are targeted – those who are not just poor, but poor for a reason (not of their own making). Crivello and Chuta (2012) cite the Ethiopian government’s definition: ‘A vulnerable child is a child who is less than 18 years of age and whose survival, care, protection or development might have been jeopardized due to a particular condition’ (p. 538). Poverty alone is inadequate. Targeting is doubtless more manageable for funders and implementers of interventions than trying to improve the situation of all poor children.^6^ The task is simply to bring orphaned children to the same living standards as non-orphans – which is clearly a less demanding task than resolving poverty.

## Inadvertently reproducing a fairy tale: The complicity of childhood studies?

Interwoven with policy agendas is research, which often finds funding and seeks to make ‘impact’ in and through the same organisations that fund interventions. Below, I outline first how academic research is at times distorted by those seeking support for their own agendas, and then, more significantly, the problems that arise from the nature of the research that has been undertaken in relation to children in southern Africa.

Meintjes and Giese (2006) have critiqued the ‘spinning of evidence’ in agency reports, alongside the use of a language of drama. They point to the ways in which sweeping statements are made without provision of evidence; measured research reports are re-articulated as definitive fact; small, non-representative localised studies are used to support generalised statements; data are not disaggregated to reveal differences between countries and regions; contradictory evidence is not mentioned; and differences between orphans and non-orphans are referred to with no indication of their magnitude. They also suggest that research itself has at times compounded the problem. Much research concerning children affected by AIDS has targeted only orphans. An earlier project of our own (Ansell and Young, 2002, 2004) is cited; this study did in fact recognise that AIDS impacts children in ways other than orphanhood, but it did not involve children deemed ‘unaffected’.

The reasons researchers focus on the impacts of AIDS on children reflect many of the contextual factors noted above. Orphanhood, from a Western perspective, is an alarming aberration from expectations of childhood. There is a large amount of funding available associated with AIDS; it is a particularly useful source of funding for children-focused research. It is also likely that the emphasis on ‘impact’ in funding decisions and in the assessment of research quality has encouraged researchers to work in areas that are of interest to policy makers and service providers.

The point I want to argue in this article, however, is that researchers are drawn to AIDS-affected children, and orphans in particular, because they are viewed as particularly illustrative of children’s social agency. I will mention a few recent examples. Abebe (2010) is critical of the way in which AIDS orphans are often cast as ‘burdens’ and in response explores how they both seek spaces of care within and make contributions to the livelihoods of their extended families. Evans’ (2011) research on sibling-headed households explored how young people expressed their agency. Payne (2012) used research with child-headed households to theoretically extend notions of agency beyond coping, resilience and competency to such children’s ‘everyday agency’. Van Der Brug (2012) highlights the agency of OVC in Namibia. Skovdal et al. (2009) argue that young carers need to be seen as social actors, and to draw policy makers’ attention to their active roles. Skovdal and Campbell (2010) argue the need to acknowledge orphans’ active coping and resilience. Skovdal and Daniel (2012) emphasise the need for policy makers and practitioners to make use of a conceptualisation of resilience as an outcome of AIDS-affected children’s agency and interactions with their social environments. These papers are merely the latest in a trend that extends back to the early years of the African pandemic and includes writing of our own (e.g. Ansell and van Blerk, 2004; van Blerk and Ansell, 2006).

The celebration of children’s agency through such research is not only a reflection of the realities of the lives of the children concerned, but is also, ironically, intended to contest the representation of AIDS-affected children as vulnerable and passive. Seeking to elaborate a key tenet of the ‘new social studies of childhood’ (James et al., 1998) – that children’s social agency has been overlooked and needs to be resuscitated – researchers seize on the example of AIDS orphans to highlight the resilience and competency that children exercise, even in the face of apparent disaster. Although not the first category of child to receive this treatment (street children were similarly feted in the 1990s), AIDS-affected children commonly take an active role in their households (as carers or supplementing livelihoods, or even heading households where adults are absent or incapacitated) and may be able or required to take decisions for which most children would depend on adults. Their agency is thus very visible and amenable to study.

While the orphan may represent the ‘quintessential vulnerable child’, it is also the hero child-as-agent of Western mythology. Countless fairy tales have an orphan as their central character (think Cinderella, Snow White, Dick Whittington), succeeding against all odds, in face of a wicked adult society.^7^ Orphans also feature in classic fiction (notably works by Charlotte Brontë, Charles Dickens, Mark Twain, Thomas Hardy, J.R.R. Tolkien) and almost all comic book superheroes are orphans (Wikipedia, 2013). Such children are depicted as unusually independent. As Kimball (1999) points out, ‘Because orphans are without the natural protection of family, they must stand on their own to conquer their problems’ (p. 564).

Childhood studies research with AIDS-affected children echoes these fictional depictions in focusing on their social agency. In so doing, it has simultaneously undermined the stereotype of the vulnerable orphan and contributed to a body of examples of children’s agency.^8^ While challenging the stereotype is doubtless valuable, the attention afforded to AIDS-affected children is problematic for the reasons outlined above: it deflects attention from the poverty that is experienced by many non-orphans and risks commodifying those who are orphaned. It also, I will argue, serves the same agendas as the policy focus on orphans that are set out above.

Returning to the fairy tale orphan, s/he has three further characteristics that recur in research accounts: s/he is both separate and different from the rest of society, and s/he acts alone (Kimball, 1999). First, far from the quintessential vulnerable child, in folktales and fiction ‘The orphan is the quintessential outcast, operates in isolation, and thus makes the perfect hero figure’ (Kimball, 1999: 561). Uncomplicated by relationships with parents, in stories orphans can act independently. This absence of restrictive ties is convenient, not only for those constructing fiction, but also in the production of research accounts of children’s agency. As is increasingly recognised, however, it does not reflect the significance of familial relationships for contemporary African orphans (e.g. Evans, 2011).

Second, fairytales represent orphans as fundamentally different from their peers – an ‘eternal other’ (Kimball, 1999). This inherent difference has perhaps been less prominent in research accounts (indeed, one is expected to extrapolate from the demonstrated agency of orphans to the agency of children more generally). Nonetheless, the idea that orphans are different probably affects the way in which such accounts are read. As noted above, orphaned children in southern Africa do not live dramatically different lives from other children, but research that singles them out by default perpetuates the idea that they do.

Finally (and relatedly), fairytale orphans usually act alone. They are profoundly independent and isolated individuals. This emphasis on the individual hero fits tidily with the neoliberal ideal of autonomy, and for this reason, research that celebrates agency can serve a neoliberal agenda as much as the policy approaches outlined above. Focusing on the ways in which the freedoms children exercise are discursively constructed in policy and research, a number of authors have suggested that the focus on children as social actors is just another historical construction of childhood, related to the international economy and the free market’s need for autonomous entrepreneurial individuals or rational unitary subjects. Kaščák and Pupala (2013), for instance, point out that childhood studies and neoliberal education discourse share the image of an active autonomous child. These discourses extolling personal autonomy also characterise interventions for AIDS-affected children (Ansell, 2010). The association between the paradigm of children’s agency and neoliberalism has also been explored by Vandenbroeck and Bouverne-De Bie (2006), who associate the phenomenon with globalisation.

This neoliberal focus on autonomy is problematic because, like the focus on orphanhood as a technical question, it shifts attention away from the structural causes of hardship. Vandenbroeck and Bouverne-De Bie (2006: 128) refer to Scott Lash (1994), whose ‘reflexive modernization thesis assumes the progressive freeing of agency from structure’. Focusing on children’s agency, as Alderson (2013) suggests, results in neglect of social structures, a situation exacerbated as children appear to be absent from structures. Researching agency also invites the use of child-centred research methods that detract attention from social structures (Alderson, 2013; Ansell, 2009). Moreover, a focus on agency can work to silence some groups, including those who do not enact it so prominently (Vandenbroeck and Bouverne-De Bie, 2006). Thus, both poverty and poor children who are not orphans are less likely to receive attention from research where the interest lies primarily in children’s agency.

## An example: Secondary school bursaries

To draw together the previous two sections, I return to the data from our research to explore the outcomes of a policy focus on orphanhood. Once orphanhood is defined as a significant problem, interventions targeted towards orphans are required. Targeting is a means of addressing poverty as an individualised issue that affects some within a community, but not others, and which can be addressed on an individual basis.

In both Malawi and Lesotho, a substantial share of the finance devoted to AIDS orphans has been invested in the provision of bursaries for secondary education. Such bursaries are provided by philanthropic organisations (in Malawi) or by the World Bank and bilateral donors via the Ministry of Education and Training (in Lesotho).^9^ The decision to focus funding on education reflects a number of assumptions: that orphans are less likely to enrol (or remain enrolled) in secondary school than other children; that the economic cost of education is an obstacle for them; and most significantly, that failure to receive an education will disadvantage them, and perhaps wider society, in the future.

The assumption is that orphans are different – less ‘advantaged’ than other children. School attendance will build their human capital and enhance their autonomy. It is, however, a highly individualised solution that relates only to those targeted and which casts responsibility for the future onto the individual. Perhaps more than other forms of neoliberal social policy, education systems are geared to the production of autonomous, individual neoliberal subjects (Liu, 2008). Vandenbroeck and Bouverne-De Bie (2006) have observed that with globalisation and neoliberalisation, a host of social problems tend to be educationalised and individualised.

Although, assisted in many cases by bursaries, the AIDS-affected young people in both case study villages had on average pursued their education further than those we deemed unaffected, none of those still resident in the villages had been able to employ that education to their own advantage. While we do not have information concerning young people from the Malawian village who were resident elsewhere, evidence from Lesotho suggested that those with secondary education were no more likely than their peers to find lucrative employment outside their own communities. Thus, although orphans are increasingly accessing schooling, schooling itself does not appear to have mitigated their disadvantage. Rather than addressing social injustice, bursary schemes incorporate orphans into a mechanism that functions to filter off a very small occupational elite but neglects the interests of the majority of poor children (Ansell, 2002). While quantitative studies suggest significant positive correlations between years of schooling and markers of individual prosperity, these are likely to be highly skewed by the small minority whose educational success affords them access to relatively very highly paid employment.

## Conclusion: Refocusing on childhood poverty through a social justice lens

The purpose of this article is not to downplay the trauma and hardship that many children face as a consequence of orphanhood, but rather to highlight how the trauma and hardship many children face for other reasons are currently obscured by a narrow focus of both research and policy on orphanhood. I have argued that the reason for this narrow focus relates in part to the tendency through the duration of the AIDS pandemic for those engaged in childhood studies to frame their research in relation to the concept of agency or the idea that children are social actors. While this lens has highlighted important aspects of the lives of such young people, it has failed to shed so much light on children who less obviously embody agency. It has also supported interventions that fail both to address the structural causes of widespread child poverty and to remediate any disadvantage experienced by individuals as a consequence of their orphan status. Thus not only does such research draw further (arguably undue) attention to AIDS orphanhood, but it also, inadvertently, advances a neoliberal agenda, locating poverty in the individual and proposing ‘solutions’ that separate out categories of youth and focus on expanding individual autonomy.

What type of research is required if we are to more adequately understand the pervasive poverty experienced by young people across the southern African region? I would argue that rather than honing in on young people’s agency, research should adopt a social justice lens to examine the contextually situated processes through which poor southern African children are systematically oppressed (see Ansell, 2014). Young (1990) suggests injustice is produced through exploitation, marginalisation, powerlessness, cultural imperialism and violence. These processes impinge on children’s lives in ways that may relate to AIDS orphanhood, but they also shape the lives of many non-orphaned children. They are inherently political and suggest a need for systemic or structural change, rather than the targeting of particular groups or support for children’s exercise of individual agency. Significantly, attending to these processes might demand a different methodological approach that is less reliant on the voices of children themselves. Children are often able talk about their experiences of orphanhood, but are perhaps less able to recount their experiences of poverty to a researcher, particularly where these do not distinguish them from other children in a community. It is also noteworthy that poverty may be more stigmatised than orphanhood, further limiting the possibilities for research that relies on children’s testimony.

## Notes

1. Co-investigators on the project were Dr Elsbeth Robson (University of Hull) and Dr Lorraine van Blerk (University of Dundee). Much of the fieldwork was undertaken by Dr Flora Hajdu (Swedish Agricultural University).

2. Because AIDS is seldom discussed openly, it was not possible to identify which illnesses and deaths were AIDS related. It is noteworthy that this very common inclusive definition of ‘AIDS-affected’ overplays the influence of AIDS and downplays the significance of other causes of ill health.

3. It is noteworthy that as many as 5% of UK children lose a parent by the age of 18 (Akerman and Statham, 2011).

4. Compare these figures with the total bilateral aid provided by DAC donors for spending on health in 2011 of $4.9 billion (OECD, 2013).

5. NGOs, UN agencies and governments often have a sharper analysis of the situations in which they seek to intervene. Aware of donor agendas, they nonetheless seek to implement policies that have wider benefits. The Malawian government, assisted by UNICEF, has been able to use Global Fund money to establish a cash transfer scheme that targets the poorest households, and not only those affected by AIDS.

6. Cheney (2010b) points out that with the shift in rhetoric from ‘orphans’ to ‘vulnerable children’, around 90% of Ugandan children fit the definition of ‘vulnerable’, a proportion that exceeds the perceived capacity of governments and others.

7. Folktales from non-Western traditions often deploy similar imagery (Kimball, 1999).

8. Street children research in the 1990s similarly saw such children as able to exercise more choice than was possible in other environments (Connolly and Ennew, 1996).

9. For a detailed discussion of Lesotho’s bursary schemes, see Ansell (2010).
